# Progress in Sympathetically Mediated Pathological Pain

**DOI:** 10.24015/JAPM.2015.0029

**Published:** 2015-06-06

**Authors:** Si-Si Chen, Jun-Ming Zhang

**Affiliations:** Pain Research Center, Department of Anesthesiology, University of Cincinnati College of Medicine, Cincinnati, USA.

## Abstract

**Aim of review:**

Many chronic pain conditions remain difficult to treat, presenting a high burden to society. Conditions such as complex regional pain syndrome may be maintained or exacerbated by sympathetic activity. Understanding the interactions between sympathetic nervous system and sensory system will help to improve the effective management of pathological pain including intractable neuropathic pain and persistent inflammatory pain.

**Method:**

We first described the discovery of abnormal connections between sympathetic and sensory neurons. Subsequently, the functional roles of sympathetic sprouting in altered neuronal excitability and increased pain sensitivity were discussed. The mechanisms of the sympathetic sprouting were focusing on its relationship with neurotrophins, local inflammation, and abnormal spontaneous activity. Finally, we discussed clinical implications and conflicting findings in the laboratory and clinical research with respect to the interaction between sympathetic system and sensory system.

**Recent findings:**

The findings that sprouting of sympathetic fibers into the sensory ganglia (dorsal root ganglion) after peripheral nerve injury, offers a possible explanation of the sympathetic involvement in pain. It is also suggested that releases of adenosine triphosphate (ATP), in addition to norepinephrine, from sympathetic nerve endings play important roles in sympathetic-mediated pain. New evidence indicates the importance of sympathetic innervation in local inflammatory responses.

**Summary:**

Hopefully, this review will reinvigorate the study of sympathetic-sensory interactions in chronic pain conditions, and help to better understand how sympathetic system contributes to this serious clinical problem.

## Sympathetic Innervation and Pathological Pain

Traumatic injury to soft- tissue, bone, and/or nerve sometimes leads to a chronic pathological pain state, known as complex regional pain syndrome (CRPS) that is characterized by ongoing pain with associated allodynia and hyperalgesia (1). Intriguingly, in some patients the pain and hyperalgesia are maintained by efferent sympathetic activity and circulating catecholamines (sympathetically maintained pain, SMP) (2), and may be partly responsive to sympathetic blockade or by administering adrenoceptor blockers, while in others the pain is sympathetically independent (sympathetically independent pain, SIP) (3). Although sympathetic blockade or sympathectomy is commonly used in the treatment of CRPS, there are actually few clinical trials on which to base this practice (4). Of note, a study reviewing extensive clinical experience with these CPRS treatments showed that sympathetic blockade is much more effective in patients with a shorter interval between the precipitating injury and the sympathectomy (or at an earlier stage of CRPS) (5). This is of interest because preclinical studies (see below) often showed that abnormal sympathetic sprouting into the dorsal root ganglion (DRG) is reduced at later stages of many pain models, or that the sympathetic influence on sensory neurons shifts to become less excitatory at later stages.

Patients with SMP or SIP often present with similar signs and symptoms (1, 6). Clinically, SMP is a component of many various neuropathic pain conditions, not only CRPS, but also phantom pain, neuralgias, herpes zoster, and fibromyalgia (FM) (7). The concept of FM as a SMP syndrome is based on the controlled studies showing that patients with FM display signs of relentless sympathetic hyperactivity and that the pain is submissive to sympathetic blockade and is rekindled by norepinephrine (NE) injections (8, 9). Sympathetic nervous system is also involved in non- neuropathic pain conditions such as temporomandibular disorder (TMD) (10), arthritis (11), and back pain (12).

### Abnormal Sympathetic Fiber Outgrowth or Sprouting in Animal Pain Models

**Figure 1:**
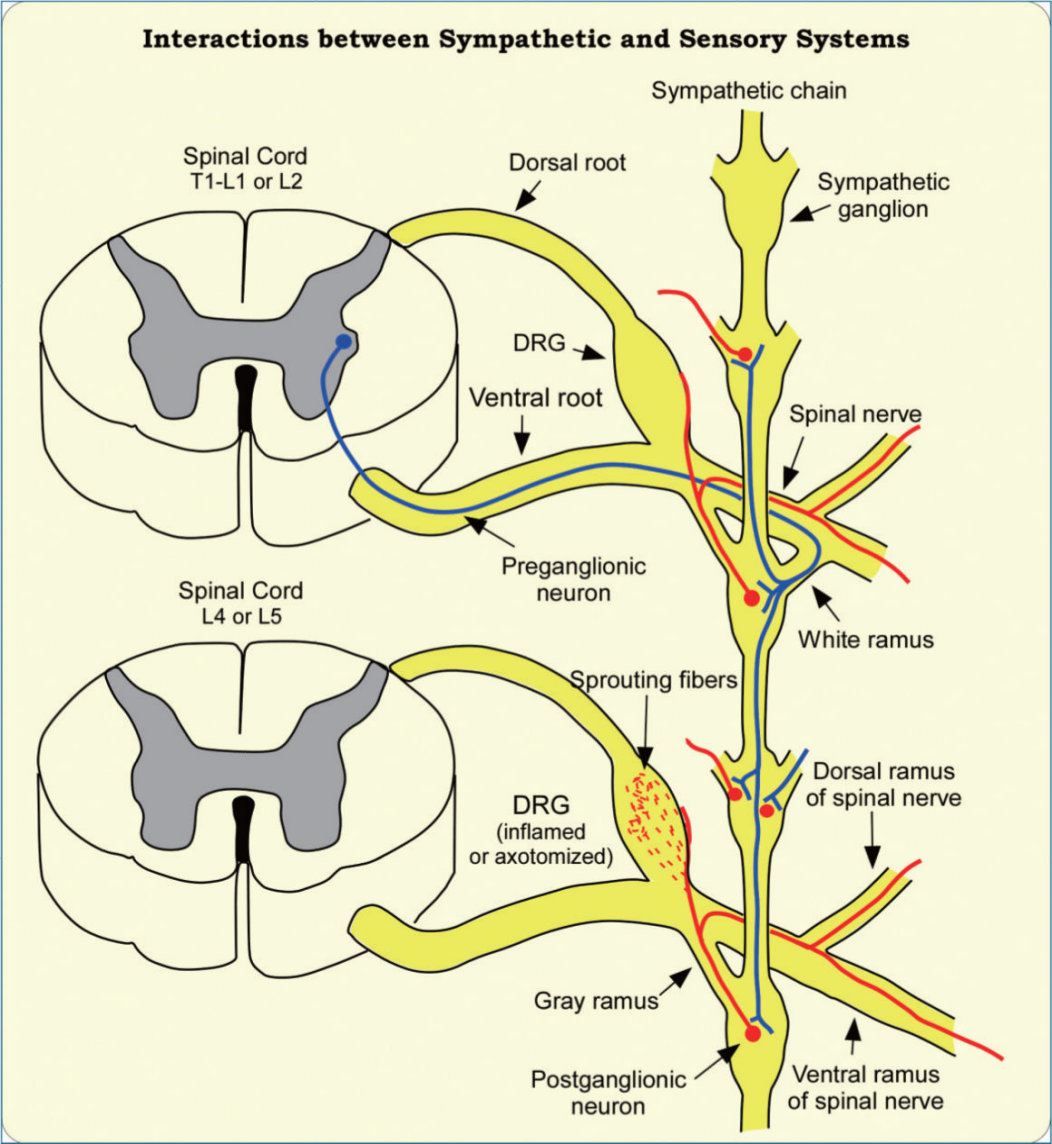
A Schematic Diagram Showing the Relationship between Sympathetic System and the Sensory System under Physiological and Pathological Conditions The preganglionic sympathetic neurons originate from the lateral dorsal horn and project to the sympathetic ganglia via the white communicating rami at T1-T12, and L1-L3 levels. Sympathetic fibers from the postganglionic neurons join the peripheral nerves via the gray rami. Note that there is no white ramus at the L4 or L5 spinal levels. After nerve injury or local inflammation, sympathetic fibers sprout from the surface of the DRG and penetrate into the ganglion, where a “basket” like structures may be formed around certain sensory neurons.

**Figure 2:**
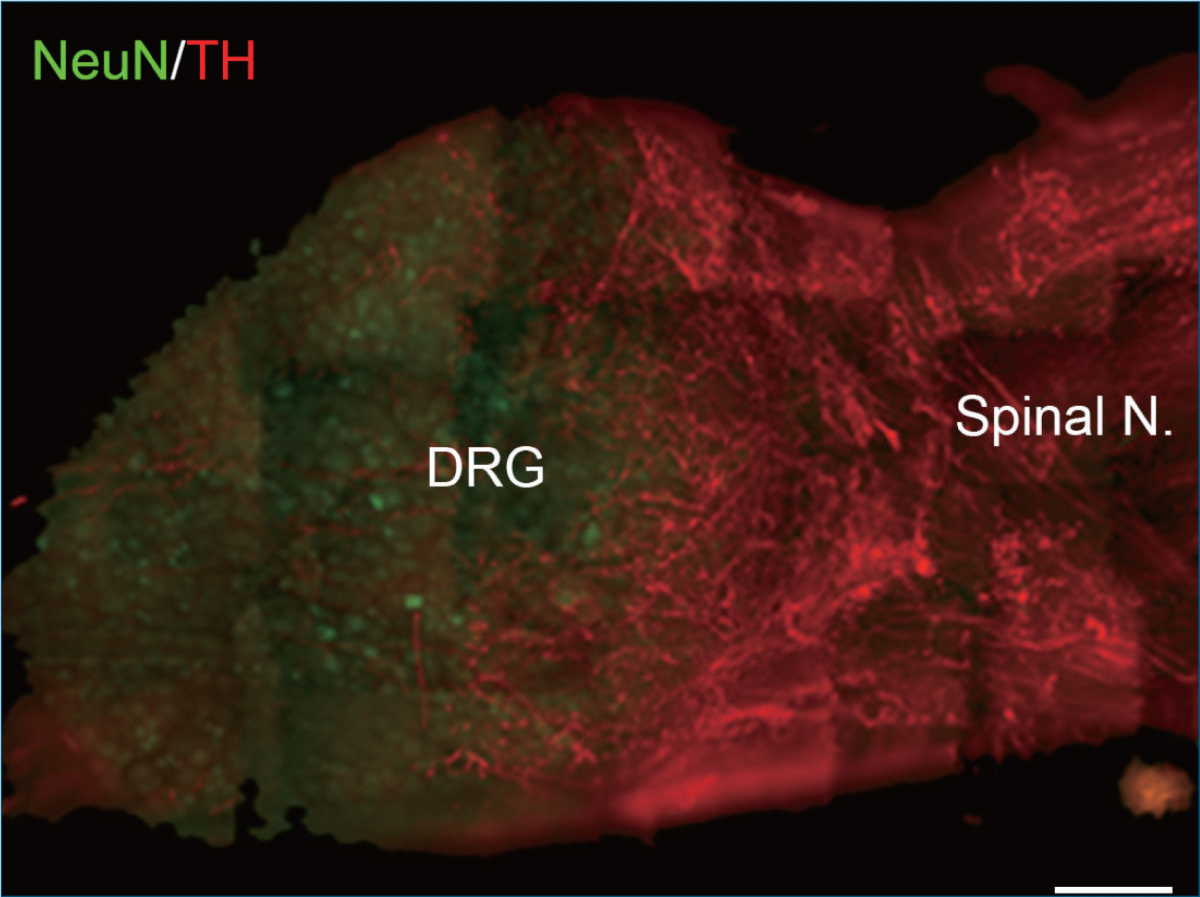
Composite Whole-Mount Staining of Tyrosine Hydroxylase (TH)-Positive Sympathetic Fibers in a DRG 3 Days after Spinal Nerve Ligation Neurons are stained with NeuN (green), and tyrosine hydroxylase is in red. Note that extensive TH-fiber sprouting onto the surface of the DRG in the SNL DRG. Scale bar: 200 μm.

Normally, the sensory neurons in the DRG are not directly innervated by the sympathetic nervous system (Figure 1). In DRG sections the only sympathetic fibers observed are associated with blood vessels, though additional fibers can be seen on the surface in whole mount preparations (13). McLachlan et al. (14) first described abnormal sprouting of sympathetic fibers into the DRG after sciatic nerve transection. Subsequent studies showed that such sprouting occurs in many animal pain models including spinal nerve ligation (SNL), chronic constriction injury (CCI), spared nerve injury (SNI), and partial sciatic nerve injury (PSNI) (15-19) (Figure 2). In a recent study, we found that three days after ligation of the ventral ramus of the spinal nerve, sympathetic fibers sprouting into the DRG were observed to originate largely in the intact dorsal ramus of the spinal nerve (20), which at the lumbar level is a small branch of the spinal nerve separating from the ventral ramus near the inter-vertebral foramen. The sprouting may include formation of dramatic “basket” formations in which sympathetic fibers form a dense plexus around individual somas (particularly of large diameter cells), and/or an increase in overall sympathetic fiber density in the cellular region of the DRG. Basket structures have also been observed in DRG from human neuropathic pain patients (21). Sprouting may occur from the fibers that normally innervate the blood vessels or as collateral fibers from sympathetic fibers that enter the spinal nerve from the grey ramus (17, 21, 22). Sprouting occurs near both intact and injured cells (23). Sprouting can occur rapidly in the absence of any axotomy, if local inflammation is induced in the DRG (24) or the DRG is compressed (25).

The timing of sprouting varies considerably from model to model, but in general the closer the peripheral nerve injury is to the DRG, the more rapidly the sprouting occurs (26). For example sprouting is observed within the first few days in the SNL model but is much slower after CCI (16) or sciatic nerve transection and SNI models. It is possible that inflammatory messengers rather than distance per se may partially explain this. As noted above, inflammation of the DRG per se induces both rapid sprouting and rapid upregulation of proinflammatory cytokines. In rats, sprouting is more rapid in the CCI model than it is after complete nerve tran-section the same distance away (17). This may be because, after CCI (but not complete transection), intact axons are mingling with severed axons distal to the injury, in which Wallerian degeneration (including macrophage infiltration) is occurring. Inflammatory mediators produced at the more distal location may be retrogradely transported back to the DRG. In support of this idea, sprouting fails to occur after CCI in transgenic mice that are deficient in Wallerian degeneration, though it does occur after SNL in these mice (27). The literature also suggests that the sprouting observed with more distal injuries, though slower, may be much more persistent than the rapid sprouting observed after proximal or inflammatory injuries.

Normally, each sensory neuron soma in the DRG is ensheathed by a single layer of satellite glia cells. After inflammation or peripheral injury these cells are activated and may proliferate and form a sheath of several layers. Sprouting occurs around neurons that are surrounded by activated satellite glia cells (28, 29). A morphological study showed that sympathetic fiber sprouts were located just outside the satellite glia layer (21). In contrast, Chung et al. (30) reported that sympathetic endings could be found directly adjacent to sensory neuron bodies. It is not clear whether this reflects a difference in the pain models used, or if the latter events are simply relatively rare, as the second study was not as quantitative as the first one. However, the satellite glia sheath permits even relatively large molecules to reach the sensory neuron, so it is generally assumed that transmitters released from sympathetic sprouts could reach the sensory neuron soma in either case. Sympathetic fibers in the DRG in both studies are shown to have synaptic varicosities and vesicles, indicating that they are likely to be functional (see below). Results from our studies suggested that sensory neurons can be influenced by diffused sympathetic fibers and by basket formations around nearby cells. Neurons with basket or ring formations are not the only neurons influenced by the sympathetic sprouting (13).

In this discussion, we have focused on sympathetic sprouting within the DRG. However it should be noted that abnormal interactions between sensory neurons and sympathetic nerves may also occur more distal to the DRG in various animal pain models.

### Mechanisms of Abnormal Sympathetic Fiber Outgrowth

Most studies of mechanisms inducing sympathetic fiber outgrowth or sprouting in the DRG have focused on the role of growth factors. Though sensory neurons are not all dependent on nerve growth factor (NGF) or related neurotrophins (NT) such as NT3 for survival in adulthood, they are still regulated by neurotrophins. In the normal DRG, NGF derived from target tissues is retrogradely transported to the cell somas. After axotomy, this transport is disrupted and NGF levels initially fall. Activated satellite glial cells (SGC) begin to synthesize NGF (and related growth factors) and provide it to the neurons, though this replacement is incomplete. This locally produced NGF has been proposed to play an important role in sprouting within the DRG; NGF potently induces sprouting in sympathetic neurons in vitro. Sprouting in DRG can be reduced by applying NGF or NT-3 antibodies within the DRG, and can be induced in uninjured DRG by overexpression of NGF in the glial cells (31-33). Glial derived neurotrophic factor (GDNF) and brain derived neurotrophic factor (BDNF) may have similar effects, possibly through a common pathway (34- 36). The proinflammatory cytokine interleukin (IL)- 6, which increases in the DRG in several pain models, has also been implicated in sympathetic sprouting, as has the cytokine leukemia inhibitory factor (LIF). These cytokines may induce sprouting primarily by inducing neurotrophin production in satellite glia cells. However, cytokines can induce sympathetic neurons to sprout in vitro, and some evidence suggests synergistic interactions between neurotrophins and cytokines, so IL-6 and LIF may also induce sprouting by direct effects on sympathetic neurons (37). In previous studies using the SNL model (38, 39), we showed that systemic or local administration of a commonly used anti-inflammatory corticosteroid, triamcinolone acetonide (TA), could reduce sympathetic sprouting, mechanical pain behavior, cytokine and NGF production in the DRG, and incidence of spontaneous bursting activity. TA may reduce sympathetic sprouting by suppressing activation of satellite cells in DRG and suppressing inflammation at the injury site or the axotomized DRGs.

### Abnormal Neuronal Activity and Sympathetic Sprouting

Abnormal spontaneous activity of sensory neurons is a feature of both neuropathic and inflammatory pain models (24, 40-44). This activity occurs very early after injury or inflammation, and blocking this activity during the initial period can reduce or prevent pain behaviors from ever developing (45- 51). Spontaneous activity also plays a key role in sympathetic sprouting in the DRG. We have found that, in several different models, early blockade of sensory neuron activity greatly reduces sympathetic sprouting, while increasing the activity increases sprouting. The bursting pattern of activity may be particularly closely associated with sprouting. Early nerve blockade also reduces satellite glial cell activation in two different pain models. Given that these cells become a source of NGF, this provides one plausible mechanism linking spontaneous activity and sympathetic sprouting. We also reported that the interaction between spontaneous activity and sympathetic sprouting could be highly localized: in rats, cells with adjacent sympathetic sprouts had higher excitability and higher incidence of spontaneous activity than other neurons in the same DRG. In transgenic mice in which sympathetic sprouts could be identified in live tissue by expression of green fluorescent protein, neurons with sympathetic baskets were much more likely to exhibit spontaneous bursting activity (19). The notion of activity-dependent sympathetic sprouting is further evidenced by the finding that knockdown of the sodium isoform 1.6 (NaV1.6) effectively reduced abnormal spontaneous activity in axotomized DRG along with decreased sympathetic sprouting (52).

#### Adenosine Triphosphate (ATP) Release/ATP Receptors in the DRG and Sympathetically Mediated Pain

In recent years much has been learned about the importance of the satellite glia in pathological pain models. There are a number of plausible mechanisms by which sensory neurons, satellite glia cells, and adjacent sympathetic sprouts might have reverberating, mutually excitatory interactions. ATP may serve as a transmitter in such positive feedback loops.

Some studies have shown that systemic blockers of various ATP channels (or genetic knockout in mice of specific P2X receptors) can reduce pain behaviors in several rodent pain models (53-56). Other studies found that repeated stimulation of the dorsal ramus which contains significant amount of sympathetic fibers enhances sensory neuron excitability, an effect that was reduced or eliminated by a “cocktail” of antagonists of NE and ATP receptors, by pretreatment with the sympathetic release blocker bretylium (20).

Within the DRG (especially after nerve injury or inflammation), ATP can be released by sympathetic fibers as a co-transmitter, can be released by SGC, and can be released from the soma of sensory neurons via activity-dependent mechanisms (57-60). Neurons express ionotropic receptors for ATP, primarily P2X2 and P2X3 receptors (in small and medium cells) or P2X5 and P2X6 receptors (in large cells) (61), which have generally excitatory effects when activated by ATP (62, 63). SGC are the only resident cells in the DRG that express P2X7 receptors (64). Activation of these P2X7 receptors can cause further ATP release by the SGC. When activated by high local concentrations of ATP (such as may occur at neuronal somatic release sites), these receptors can (in combination with other proteins) form very large pores that may mediate release of cytokines, including tumor necrosis factor (TNF)-α and IL-1β (64, 65). These cytokines in turn may cause additional excitatory effects on neighboring neurons. TNF-α in particular can potentiate P2X3- mediated responses. Sympathetic fibers express several autoreceptors for ATP, some of which further increase transmitter release (66). Hence there are multiple ways in which abnormal activity in sensory neurons might lead to further excitation of sensory neurons, via ATP-dependent mechanisms involving neurons, sympathetic fibers, and glial cells.

Many of the excitatory pathways activated by extracellular ATP are present in normal DRG. For example, activity-dependent somatic release of ATP by sensory neurons occurs normally, and most P2X type ATP receptors are present in normal DRG. However, many of these excitatory pathways are enhanced after nerve injury or inflammation (67). Examples include upregulation of P2X2 and P2X3 receptors after chronic compression of DRG (CCD) (68), upregulation of P2X3 receptors in the formalin model (69), sensitized P2X responses after SNI (70), increased P2X receptor expression and sensitivity after SNL (71, 72), the increase in ATP release by glial cells after activation, increased abnormal activity of sensory neurons which should increase somatic ATP release, the abnormal sprouting of sympathetic fibers that release ATP along with NE, the potentiation of P2X2/3 responses by NE (73), and the increase in baseline ATP release from DRG after sciatic nerve entrapment (74). Thus the excitatory roles of ATP may be especially important in pathological states, a concept which has also been proposed in the central nervous system (CNS) (67).

Many of the mechanistic studies on somatic release of ATP from sensory neurons cited above have been conducted on small and medium cells. In addition, many studies of ATP responses have been conducted in small and medium cells. This may reflect a focus on nociceptors, and/or the greater ease of maintaining small and medium cells in primary culture. In contrast, studies of spontaneous activity have focused on medium and large cells. Large low-threshold cells are generally presumed to play important roles in allodynia, and they are also the predominant sites of sympathetic basket formation. However, the initial studies on cross-excitation in DRG neurons, which first suggested the possibility of neuronal release of diffusible local messengers within the DRG, were conducted in Aβ neurons (75, 76), suggesting that somatic release also occurs from large neurons. There is also the possibility of interactions between different neuron types within the DRG, either through local paracrine actions (77), coupling of SGC around neighboring neurons (78), or through peptidergic sprouting of nociceptors around large cells within the DRG (29). This could be one explanation for the observation that sensitization of P2X3 receptors, found primarily in nociceptors, contributes to allodynia even though other lines of evidence implicate large diameter neurons that normally detect innocuous stimuli (70).

The presence of positive feedback loops and reverberating excitatory pathways may help explain why sympathetic blockade (in animal models and in human patients), as well as blockade of abnormal sensory neuron activity in animal models, may have antinociceptive effects that far outlast the duration of the blockade. Such systems can be highly nonlinear in their behavior.

#### Proinflammatory Effects of Sympathetic Innervation

Above we have discussed mechanisms by which inflammation at the level of the DRG promotes sprouting of sympathetic fibers. However, it is also plausible that the sympathetic fibers sprouting into the DRG might further promote inflammatory processes there, creating another positive feedback loop. For example, ATP release could promote activation of SGC due to the excitatory receptors expressed on these cells. Sympathetic excitation of sensory neuron activity could also promote glial activation as well as directly enhancing release of proinflammatory cytokines from neurons. Sympathetic fibers can be activated by inflammation, and the NE, ATP, neuropeptide Y (NPY), and adenosine thereby released can then have profound effects on immune cells that mediate the inflammatory response as well as on the changes in blood vessel endothelium that allow these cells to enter the affected tissue. NE effects may be proinflammatory or anti-inflammatory, depending on the receptor types and immune cell types involved and on the actions of cortisol. ATP effects on immune cells are predominantly proinflammatory, while those of adenosine are predominantly anti-inflammatory. The local concentrations of these neurotransmitters also help determine which type of effect predominates (79, 80). In several conditions in which this has been studied (e.g., joint inflammation and gut inflammation), the sympathetic system is proposed to play a largely proinflammatory role in the early phases and a largely anti-inflammatory role at later phases (81, 82). This has some intriguing parallels to the finding of inhibitory effects of sympathetic sprouts at later stages of the complete sciatic nerve transection (CSNT) model (83), and to the relative ineffectiveness of sympathectomy in CRPS at later stages of the disease (5).

Interestingly, inflammation may increase abnormal sympathetic sprouting as evidenced by reduced sprouting after local administration of anti-inflammatory corticosteroids (39).

### Function of Sympathetic Sprouts: Behavioral and Cellular Studies

By analogy with the clinical experiences in humans, the sympathetic system is predicted to exacerbate pain behaviors in animal models. This has been tested in several models by performing chemical or surgical sympathectomy at different time points relative to the establishment of the pain model, and by use of sympathetic agonists and antagonists. Such studies have had mixed results. Pertin et al. (18) reviewed much of this work (see table in reference 18). For both mechanical and thermal pain behaviors, the majority of studies showed that sympathectomy reduced pain behavior. Lee et al. (84) suggested that some of these discrepancies might be due to differences in the precise anatomical location of the hind paw pain testing. Some papers that have argued against sympathetically enhanced pain in various models have done so on the basis that sprouting and pain behaviors are not temporally well correlated. Some have shown that adrenergic agonists or antagonists did not have the expected behavioral results. Reported effects of sympathectomy are unlikely to be due entirely to changes in blood flow to the DRG, as sympathetic denervation has been demonstrated to have only very short-lasting effects on blood flow in the spinal nerve or cauda equina (85, 86).

A few studies have looked at the effects of sympathetic stimulation on sensory fiber activity, primarily in in- vivo preparations. In their original paper describing sympathetic sprouting after sciatic nerve transection, McLachlan et al. (14) showed that a short burst of activity in sensory axons could be recorded with extracellular recording methods upon stimulation of the preganglionic sympathetic fibers in vivo. In the same model, it was shown that adrenergic agonists increased spontaneous firing from DRG neurons. Effects of sympathetic activation are not always excitatory, however, Michaelis et al. (83) showed that inhibitory effects predominated at time points later after the injury.

### Limitations in the Current Understanding of Sympathetic Outgrowth and Sympathetic Components in Pathological Pain

The mixed results discussed above, especially in behavioral studies, may have led researchers to question the relevance of animal models of sympathetic effects on chronic pain. This seems to have resulted in diminished attention to this area in recent years. This is unfortunate, given the clinical importance of this issue, and we think that a further investigation of the role and mechanisms of sympathetic-sensory neurons is overdue, especially in view of recent developments in related fields. There are some important limitations to the previous research in this field, and possible explanations for some of the reported discrepancies.

The possible roles of sympathetic transmitters other than NE have often been overlooked. Although surgical or chemical sympathectomy has been proved to be effective in reducing pain in animal models and sympathetic blockade has been an effective treatment in managing pain in some CRPS patients, in vitro studies of NE on sensory neurons have been inconclusive as to why sympathetic blockade or sympathectomy should reduce pain. Many researchers have reported increased sensitivity of DRG neurons to NE in nerve injured animals but the doses in these studies (73, 87, 88) were often at *μ*M and even mM levels. The above observations suggest that neurotransmitters other than or in addition to NE are involved in SMP. It is somewhat surprising that most research has focused on NE, since the existence of sympathetic co-transmitters such as ATP and NPY has been known for many years. However, in a number of papers, the studied effects described as “sympathetic stimulation” were actually the effects of noradrenergic agonists, or the reported lack of effect of sympathetic blockade (89) was actually a lack of effect of adrenergic blockade. In particular ATP, a common co-transmitter in sympathetic neurons, can have excitatory effects on both SGC and sensory neurons (see below). In addition, ATP and NE may have synergistic effects on sensory neurons (90), especially in pain models (53, 73).

Lack of good temporal correlation between sprouting and pain behavior in some studies may be due to the existence of both excitatory and inhibitory effects of sympathetic fibers on sensory neurons, with the balance shifting towards inhibition over time. Animal models with rapid sympathetic sprouting in the DRG may not respond to sympathectomy performed later in the model, due to the well-known phenomenon of spinal cord sensitization and centralization of pain and/or to the later disappearance of sprouts from the DRG. This, however, does not negate the possible importance of excitatory sympathetic-sensory neuron interactions in initiation of pain behaviors in such models. Indeed this correlates well with some of the clinical studies on sympathetic blockade in humans.

Traditional surgical sympathectomy can be quite difficult to achieve and is a relatively invasive surgery. In some studies, “sympathectomy” may actually refer to cutting of the preganglionic fibers and connections between the sympathetic ganglia, not actual removal of the sympathetic ganglia or their connections to spinal nerves. Interestingly one study (91) suggested that while postganglionic sympathetic fibers made important contributions to pain behavior in the SNL model, this did not require the presence of the preganglionic fibers. Results from our laboratory using an isolated DRG preparation showed that a subset of sympathetic fibers sprouting into the DRG may be spontaneously releasing transmitters. We reported striking behavioral effects of a much less invasive method of blocking sympathetic components in the SNL model (in preparation).

Functional studies directly examining sympathetic-sensory neuron coupling have been very limited, and largely confined to fiber recording studies in vivo. While in vivo studies are very important, in some cases it may be difficult to separate direct effects (e.g., of sympathetic agonists) at the DRG level from systemic effects. Some of the extracellular fiber recording studies (92, 93) looked at effects of sympathetic stimulation on ongoing spontaneous activity. In such recordings it is difficult to detect a previously silent fiber that becomes active after sympathetic stimulation (since fiber bundles may be initially selected for recording based on the presence of some spontaneous activity), and other types of excitatory effects such as that action potential broadening or reduction in threshold will be missed. The functional connections between sympathetic sprouts and sensory neurons can be better studied in an isolated DRG preparation, in which sensory neurons are examined by intracellular microelectrode recording. This preparation also makes it feasible to use and interpret pharmacological probes.

## Clinical Implications

Recent studies and meta-analyses have emphasized that there are relatively few well-designed, randomized double blinded clinical trials of anti-sympathetic interventions in most of the pain conditions for which they are commonly used--these papers usually call for further research and note positive results in less rigorous or smaller trials (94-98). It is noted that pre-emptive sympathetic block has recently been successfully tested as a way to prevent chronic pain development (99), or to control postoperative pain (100), new uses consistent with our preclinical data. An interesting study (101) showed dramatic results using a catheter approach to provide longer lasting sympathetic blockade than that-achieved by injection, in 293 patients with intractable pain. Because pain relief often required more than a week of blockade, the authors noted that most patients would therefore have been traditionally classified as having SIP. It is therefore believed that there is a need for further pre-clinical research in this area that will complement the expanding clinical interest.

## Summary

Chronic pain conditions remain difficult to treat, and create a high morbidity burden to society. We have previously demonstrated that abnormal sensory neuron activity plays a key role in initiating DRG pathology including sympathetic sprouting. It is highly likely that mutually excitatory interactions between sensory neurons, SGC, and sympathetic sprouts play important roles in the initiation of the chronic pain state. It is possible that local inflammatory processes and the transmitter ATP are important components of these excitatory interactions. Local inflammation promotes spontaneous activity (especially bursting), which activates surrounding glia and induces sprouting of sympathetic nerve fibers. Activated glia and adjacent sympathetic sprouts in turn provide further excitatory inputs to the DRG neuron via the release of neurotransmitters such as ATP and further provoke the inflammation process.

For physicians to manage pathological pain, it is critical to have a complete understanding of the disease state. Pathological pain can no longer be simply classified into SMP and SIP. Based on our understanding, many pain conditions may involve the sympathetic system especially at the early stage. Consequently, sympathetic blockade may have remarkable effect, partial effect, no effect, or complete opposite effect depending on the disease state. It would be a wise approach to have a diagnostic treatment as is being performed by many pain physicians already in their treatment plan.
